# miR‐155‐regulated mTOR and Toll‐like receptor 5 in gastric diffuse large B‐cell lymphoma

**DOI:** 10.1002/cam4.4466

**Published:** 2021-12-16

**Authors:** Wei‐Ting Huang, Sung‐Hsin Kuo, Yi‐Chun Kuo, Chung‐Wu Lin

**Affiliations:** ^1^ Department of Pathology National Taiwan University Hospital Taipei Taiwan; ^2^ Department of Oncology National Taiwan University Hospital Taipei Taiwan

**Keywords:** gastric lymphoma, *H. pylori*, miR‐155, mTOR, TLR5

## Abstract

**Background:**

Gastric diffuse large B‐cell lymphoma (DLBCL) is often associated with *Helicobacter pylori (H. pylori)* infection. Those in the early stage could be treated with *H. pylori* eradication therapy, and are classified into a sensitive group and a resistant group.

**Methods:**

Genome‐wide miRNA and miRNA expression profiles were obtained from biopsy specimens of gastric DLBCL. MiRNAs and their targets as predictors of responses to *H. pylori* eradication therapy were identified through differential expression and pathway enrichment analysis, and further confirmed with transfection experiments in lymphoma cell lines of B‐cell origin.

**Results:**

Genome‐wide miRNA and mRNA profiles showed miR‐200 was associated with the sensitive group, and that the resistant group had higher levels of miR‐155 and lower levels of DEPTOR (an inhibitor of mTOR) than the sensitive group. BJAB cells transfected with miR‐155 also had lower DEPTOR and higher mTOR levels. Therefore, miR‐155‐mediated inhibition of DEPTOR with secondary activation of mTOR was a potential marker for resistance to *H. pylori* eradication therapy. In contrast, pathway enrichment analysis showed that Toll‐like receptor 5 (TLR5), the receptor for bacterial flagellin, was a potential marker for sensitivity to *H. pylori* eradication therapy. In an independent series, stronger expression of pS6K1 (a direct target of mTOR) was associated with the resistant group and morphologic evidence of active gastritis was associated with the sensitive group.

**Conclusions:**

These findings showed that activation of the miR‐155‐DEPTOR pathway is a marker for resistance to *H. pylori eradication therapy*, and that histological evaluation of active gastritis might be used as a surrogate marker to predict responses to *H. pylori* eradication therapy in gastric DLBCL.

## Graphical abstract

The miR‐155‐DEPTOR‐mTOR pathway induced resistance to *H. pylori* eradication therapy in gastric DLBCL. TLR5, the receptor for bacterial flagellin, was associated with sensitivity to *H. pylori* eradication therapy in gastric DLBCL. The presence of active gastritis and pS6K1 are surrogate markers for TLR5 and mTOR, respectively.

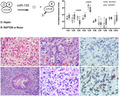

## 1 INTRODUCTION

Primary non‐Hodgkin lymphomas of the stomach include indolent MALT (mucosa‐associated lymphoid tissue) lymphoma and aggressive diffuse large B‐cell lymphoma (DLBCL).^1^ Both gastric MALT lymphomas and DLBCLs are often associated with *Helicobacter pylori (H. pylori)* infection.^2^, ^3^ Early stage gastric MALT lymphoma has been treated with *H. pylori* eradication therapy, and more recently, some of the early stage gastric DLBCLs were also shown to be responsive to *H. pylori* eradication therapy.^4^ However, data on the molecular features that may be used to predict sensitivity or resistance to *H. pylori* eradication therapy are still limited.

The mammalian target of rapamycin (mTOR) is a serine/threonine kinase that regulates cell growth and metabolism. Normally, mTOR is a component of a multi‐protein mTOR complex 1 (mTORC1) or complex 2 (mTORC2). RAPTOR in mTORC1 and RICTOR in mTORC2 may inhibit mTOR.^5^, ^6^ In addition, DEPTOR, a regulatory protein found in both mTORC1 and mTORC2, also inhibits mTOR and prevents mTOR from activation.

mTOR may be activated directly through stimulation of the PI3K/AKT pathway or indirectly through inactivation of DEPTOR. In turn, the PIK3/AKT/mTOR pathway is regulated by microRNAs. For example, miR‐155 may inhibit PIK3R1 (p85α), a negative regulator of the PI3K‐AKT pathway in nodal DLBCLs.^7^ Similarly, miR‐142‐3p may inhibit RICTOR with secondary activation of mTOR in nasal NK‐cell lymphomas.^8^ Because mTOR may induce cellular growth and proliferation,^9^ mTOR inhibitors have been used therapeutically in the treatments of lymphoma,^10^, ^11^, ^12^ and mTOR regulatory miRNAs may have potential therapeutic applications.

Toll‐like receptors (TLRs) are pattern recognition receptors that recognize conserved molecular patterns shared by pathogens. There are 10 TLRs in humans, TLRs 1, 2, 4, 5, 6, and 10 are membrane receptors for bacterial encoded proteins or lipids, and TLRs 3, 7, 8, and 9 are found in intracellular vesicles for nucleic acids. The receptors are key players in innate immune system,^13^ including the inflammatory responses in *H. pylori* infection.^14^ In addition, TLRs are involved in the pathogenesis of B‐cell lymphomas and *H. pylori*‐associated gastric malignancies.^15^, ^16^, ^17^

Biologic markers that can be used to identify early stage gastric DLBCLs for *H. pylori* eradication therapy are clinically significant. In the present study, genome‐wide miRNA profiles showed miR‐155 as a marker for resistance to *H. pylori* eradication therapy. Furthermore, a wet lab approach plus mRNA profiles identified miR‐155‐activated mTOR pathway as a maker for resistance and a dry lab approach identified miR‐155‐centered expression of TLR5 as a marker for sensitivity. Finally, active gastritis and phosphorylated S6K1 were shown to be surrogate markers for TLR5 and mTOR, respectively, in a prospective series of early gastric DLBCLs treated with *H. pylori* eradication therapy.

## 2 MATERIALS AND METHODS

### 2.1 Tissue samples and clinical data

Stage I/II gastric DLBCLs treated with *H. pylori* eradication therapy were used in the present study, including 17 cases from a retrospective study and 44 cases from a prospective study (Table 1).

**TABLE 1:** Clinical features of stage IE/IIE1 gastric diffuse large B‐cell lymphomas treated with first‐line *H. pylori* eradication therapy

Clinical characteristics	Response to *H. pylori* eradication therapy			*p* *
	Total number (*n* = 44)	Complete remission (*n* = 21)	Non‐complete remission (*n* = 23)	
Age (median, range, years)	67 (24–100)	57.5 (34–89)	70 (24–100)	0.397^#^
Gender				
Women	25 (56.8%)	10 (47.6%)	15 (65.2%)	0.239^§^
Men	19 (43.2%)	11 (52.4%)	8 (34.8%)	
Endoscopic features, *n* (%)				
Ulceration or ulcerated mass	36 (81.8%)	15 (71.4%)	21 (91.3%)	0.088^§^
Erosions on giant nodular folds or multiple nodular lesions	8 (18.2%)	6 (28.6%)	2 (8.7%)	
Location of tumor (s), *n* (%)				
Proximal^a^ or ≥2 components	15 (34.1%)	4 (19.0%)	11 (47.8%)	0.044^§^
Distal^b^	29 (65.9%)	17 (81.0%)	12 (52.2%)	
Stage				
IE	32 (74.4%)	15 (75.0%)	17 (73.9%)	0.935^§^
IIE1	11 (25.6%)	5 (25.0%)	6 (21.6%)	
IPI score				
0	15 (34.1%)	10 (47.6%)	5 (21.7%)	0.10^§^
1	27 (61.4%)	11 (52.4%)	16 (69.6%)	
2	2 (4.5%)	0 (0%)	2 (8.7%)	

^a^ Proximal: Middle body, upper body, fundus, or cardia.

^b^ Distal: Antrum, angle, or lower body.

* *P*: comparison of discrete variables between complete remission cases and non‐complete remission cases.

# *P* values (two‐sided) were calculated using the Student's *t*‐test.

§ *P* values (two sided) were calculated using the chi‐squared test or the Fisher’s exact test.

Between January 2003 and December 2017, patients with stage IE/IIE1 *H. pylori*‐positive gastric DLBCL were treated with first‐line *H. pylori* eradication therapy, such as 500 mg of amoxicillin four times daily (or 1000 mg of amoxicillin administered twice a day), 500 mg of clarithromycin twice daily, and 20 mg of omeprazole or 30 mg of lansoprazole twice daily for 2 weeks.

At the time of initial diagnosis, six biopsy specimens from the lymphomas were taken. Formalin‐fixed paraffin‐embedded tissue blocks were used for pathologic evaluations. Sections with extensive infiltrations of diffuse large lymphoma cells were used for the expression studies. The diagnosis of DLBCL was based on the criteria of WHO classification. Lymphoma cells could manifest as clusters, confluent aggregates, or sheets of large cells.

All patients received initial staging workups, including the assessment of ECOG performance status, a physical examination of Waldeyer's ring, a detailed history, evaluation of hemogram and lactate dehydrogenase, examination of computed tomographic scan of the neck, chest, abdomen and pelvis, bone marrow assessment, or fluorine‐18 fluorodeoxyglucose positron emission examination. Endoscopic findings, such as ulceration, ulcerated mass, erosions on giant nodular folds, or nodularity, and the locations, such as cardia, body, and antrum of DLBCL in the stomach were recorded. The staging and classification of lesions were based on the Musshoff modification of the Ann Arbor staging system.

All patients underwent follow‐up upper gastrointestinal endoscopic examination 4–6 weeks after completion of *H. pylori* eradication therapy, which was repeated every 8–12 weeks until complete remission. The sensitive group achieved complete remission at a median time of 2 months. The resistant group had residual, stable, or progressive diseases.

For patients without complete remission or without partial remission via endoscopic or computed tomographic scan examination during follow‐up after completing *H. pylori* eradication therapy, we immediately administrated rituximab plus chemotherapy or chemotherapy for these patients to avoid rapid progression of DLBCL.

### 2.2 Genome‐wide miRNA profiles in gastric diffuse large B‐cell lymphomas

A series of nine *H. pylori* eradication therapy‐sensitive and eight *H. pylori* eradication therapy‐resistant gastric DLBCLs was used. RNAs were extracted from sections of the biopsy specimen taken at the time of initial diagnosis. Sections of the specimens showed more than 90% atypical lymphoid infiltrates. A custom‐designed nCounter Custom CodeSet of 654 miRNAs, submitted to NCBI as GEO GPL18086 and the nCounter miRNA Expression Analysis System (NanoString Technologies) were used.^18^, ^19^, ^20^ Data S1 were submitted as GEO Super‐series GSE182362.

### 2.3 BJAB and U2932 cell lines and transfection

BJAB and U2932 cells derived from B‐cell lymphomas were obtained from Bioresource Collection and Research Center (BCRC, Hsinchu, Taiwan) and grown in RPMI160 medium. Flow cytometry and genome‐wide mRNA expression profiles were used to confirm an origin from B‐cell lymphomas. For transfection experiments, 20–40 μg of EGFP‐expressing plasmid was electroporated into the cells, followed by selection with G418 until greater than 90% were positive for EGFP.

### 2.4 Genome‐wide mRNA profiles in miR‐155‐BJAB and miR‐200 a, b, or c‐U2932

For identification of the targets of miR‐155 or miR‐200, BJAB cells transfected with a miR‐155‐expressing vector or three U2932 cell lines transfected with an expressing vector for miR‐200 a, b, or c, respectively, were established.

Genome‐wide mRNA profiles were performed with Agilent‐072373 SurePrint G3 Human GE 8 × 60 K Microarray (NCBI GEO platform GPL21185) for miR‐155‐BJAB cells and with Agilent‐028004 SurePrint G3 Human GE 8 × 60 K Microarray (NCBI GEO platform GPL14550) for miR‐200‐U2932 cells, according to the manufacturer's recommendations (Agilent Technologies). Data S2 and S3 were submitted as GEO Super‐series GSE182362.

### 2.5 Genome‐wide mRNA profiles in gastric diffuse large B‐cell lymphomas

A series of eight *H. pylori* eradication therapy‐sensitive and eight *H. pylori* eradication therapy‐resistant gastric DLBCLs was used. RNAs were extracted from the biopsy specimen taken at the time of initial diagnosis. All the sections had >90% atypical lymphoid infiltrates. Agilent‐039494 SurePrint G3 Human GEv2 8x60K Microarray (NCBI GEO platform GPL17077) was used according to the manufacturer's recommendations (Agilent Technologies). Data S4 were submitted as GEO Super‐series GSE182362.

The Agilent arrays, −072373, −028004, and −039494, were similar, belonging to the same series of SurePrint G3 human 8 × 60 K microarrays from Agilent. Data analysis of this study did not involve cross‐platform comparisons and did not require identical arrays.

### 2.6 Luciferase activity assay for inhibition of DEPTOR by miR‐155 and localization of the binding site

Luciferase assays were performed in U2932 cells grown under serum‐starved conditions with a plasmid derived from the pmirGLO Dual‐Luciferase miRNA target expression vector (Promega). The plasmid, miRCS‐GLO‐targetCS, includes cloning sites (CSs) for both miRNA and 3'UTR of the target. The activity of firefly luciferase was the primary reporter to monitor inhibition of the targets by miRNAs and the activity of Renilla luciferase was used as an internal control for normalization.

For inhibition of DEPTOR by miR‐155, the wild‐type (wt) or mutant (mt) 3'UTR of DEPTOR, about 1.2 K, was inserted downstream the firefly luciferase coding region and the wild‐type (wt) or mutant (mt) miR‐155 stem‐loop precursor was inserted into the miR expression cassette. The resultant vector, wt‐miR‐155‐GLO‐wt‐DEPTOR‐3'UTR, mt‐miR‐155‐GLO‐wt‐DEPTOR‐3'UTR, wt‐miR‐155‐GLO‐mt‐DEPTOR‐3'UTR, or mt‐miR‐155‐GLO‐mt‐DEPTOR‐3'UTR, was transfected into U2932 cells.

Luciferase activities were measured according to the manufacturer's protocol. The ratio of firefly luciferase activity to Renilla luciferase activity was obtained and was normalized to the ratio of wt‐miR‐155‐GLO‐mt‐DEPTOR‐3'UTR.

### 2.7 Western blotting for DEPTOR, p‐mTOR, and GAPDH in BJAB cells transfected with miR‐155

The stem‐loop of miR‐155 was cloned into an EGFP+ expression vector. The following antibodies were used in western blotting: DEPTOR (rabbit monoclonal, D9F5, #1816) and p‐mTOR (Ser2448) (rabbit polyclonal, #2971) from Cell Signaling Technology, Danvers, and MA and GAPDH (rabbit polyclonal, FL‐335, #25778) from Santa Cruz Biotechnology, Santa Cruz, CA.

### 2.8 qRT‐PCR for miR‐155, U6, DEPTOR, and Actin beta

Quantitative RT‐PCR for miR‐155 or U6 was performed in total RNAs extracted from formalin‐fixed, paraffin‐embedded tissue blocks with a stem‐loop RT‐PCR. For RT, the reverse primer was: 5'‐N44‐AACCCCT‐3' for miR‐155 or 5'‐N44‐AAAAATATGGAACG CTT‐3 for U6, where N44 was 5'‐GTC‐GTA‐TCC‐ATG‐GCA‐GGG‐TCC‐GAG‐GTA‐TTC‐GCC‐ATG‐GAT‐ACG‐AC‐3'. For PCR, a universal reverse PCR primer, 5'‐TGG‐CAG‐GGT‐CCG‐AGG‐T‐3', and a miR‐155‐specific forward primer 5'‐GGG‐TTA‐ATG‐CTA‐ATC‐GTG‐AT‐3’ or an U6‐specific forward primer 5'‐ATT‐AGC‐ATG‐GCC‐CCT‐GCG‐CAA −3' were used. The PCR products had a size of 61 bp for miR‐155 and 92 bp for U6.

Quantitative RT‐PCR for DEPTOR and ACTB (beta‐actin) was performed with the following primers: 5'‐TCT‐CCT‐CAC‐TGC‐AGA‐CAC‐GAT‐3' (RT and PCR reverse primer for DEPTOR at exon 6), 5'‐CTG‐AGG‐AAG‐CAG‐AGC‐CAT‐GA‐3' (PCR forward primer for DEPTOR at exon 5), 5'‐CTG‐GAA‐GGT‐GGA‐CAG‐CGA‐GGC‐3' (RT and PCR reverse primer for ACTB at exon 6), and 5'‐CCC‐AGC‐ACA‐ATG‐AAG‐ATC‐AAG‐3' (PCR forward primer for ACTB at exon 5). The PCR products had a size of 93 bp for DEPTOR and also 93 bp for ACTB.

### 2.9 Immunohistochemistry for phosphorylated S6 kinase 1 (S6K1)

Immunoperoxidase stains were performed with an antibody against phosphorylated S6K1 (rabbit monoclonal SP50, Abcam) on formalin‐fixed paraffin‐embedded tissue sections. Antigen retrieval was performed in Tris buffer at pH 8. The primary antibody was applied to the slides at 37°C for 45 min. Biotin‐conjugated secondary antibodies, peroxidase‐conjugated streptavidin, and DAB were used sequentially to complete the reactions.^21^

### 2.10 Statistics and bioinformatics

**FIGURE 1:**
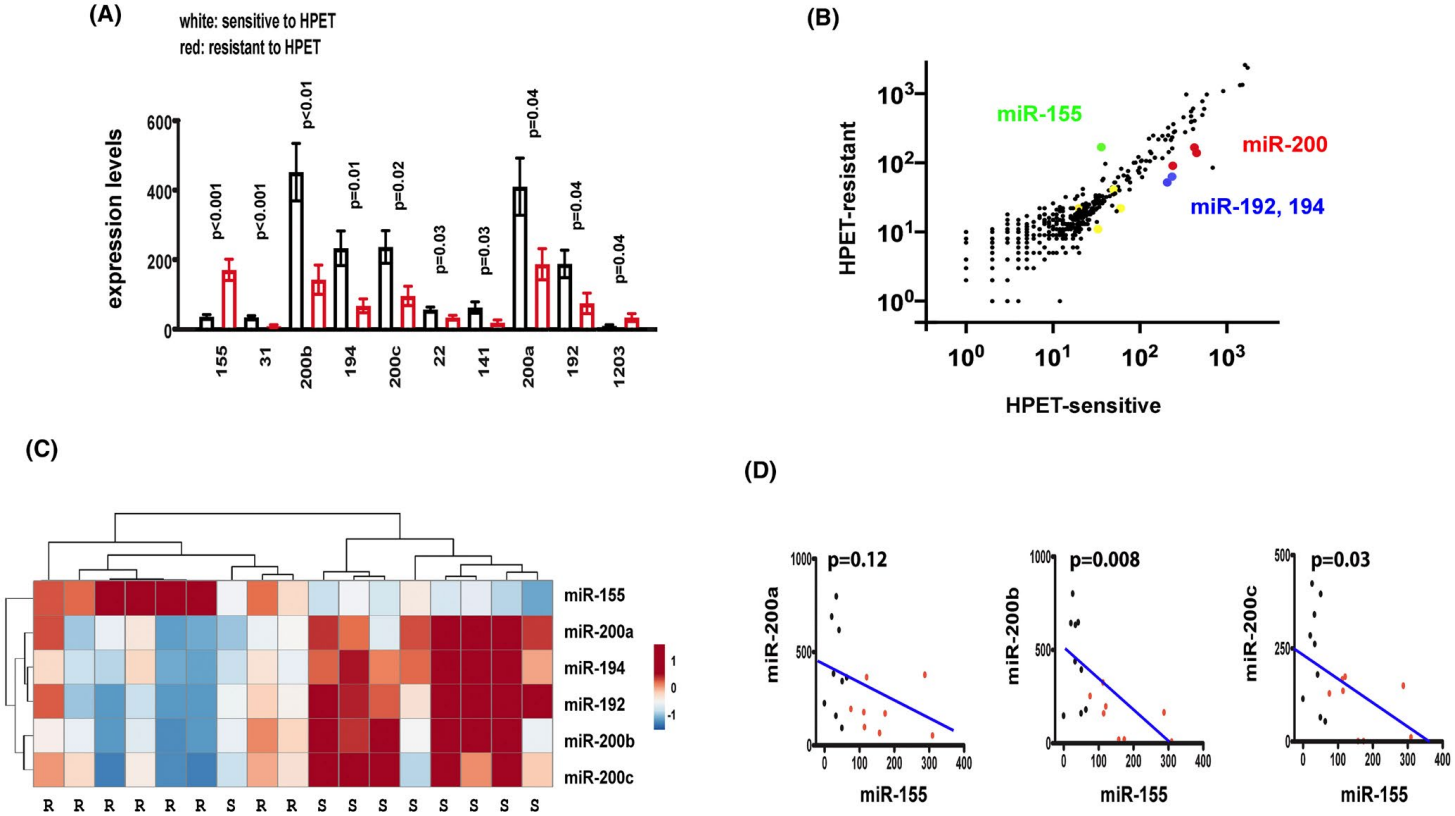
In gastric diffuse large B‐cell lymphomas treated with *H. pylori* eradication therapy, the resistant group had higher levels of miR‐155 but lower levels of miR‐200 than the sensitive group. (A) The sensitive group had lower levels of miR‐155 and higher levels of miR‐200 than the resistant group. The NanoString technology is used to determine the levels of 654 miRNAs in 9 sensitive and 8 resistant cases. MiR‐155 is the only miRNA increased in the resistant group and miR‐200 a, b, and c are the most abundant miRNAs increased in the sensitive group. X‐axis: Ten miRNAs that are differentially expressed in the sensitive versus the resistant group. Y‐axis: mean expression levels. White: sensitive to HPET; Red: resistant to HPET. HPET: *H. pylori* eradication therapy. (B) Scatter plot for the expression levels of 654 miRNAs in gastric diffuse large B‐cell lymphomas. Most miRNAs had similar levels in the sensitive and resistant groups and are clustered along the x = y diagonal. Of the 10 differentially expressed miRNAs, miR‐155 (green) is above the diagonal and miR‐200 a, b, c (red), miR‐192, and miR‐194 (blue) are below the diagonal. The remaining four minor miRNAs, 31, 22, 141, and 1203 are represented by the yellow dots. X‐axis: sensitive to *H. pylori* eradication therapy; Y‐axis: resistant to *H. pylori* eradication therapy. (C) Hierarchical clustering of gastric diffuse large B‐cell lymphomas with miR‐155, miR‐200, miR‐192, and miR‐194. The sensitive cluster on the left included one resistant plus eight sensitive cases. The resistant cluster on the right included eight resistant cases. (D) An inverse correlation between miR‐155 and miR‐200 a, b, or c. The correlation coefficient between miR‐155 and miR‐200 a, b, or c, is −0.53, −0.63, and −0.58, respectively. X‐axis: level of miR‐155; Y‐axis: levels of miR‐200 a, b, or c

Two‐sample comparisons were performed with the *t*‐test for Figure 1A, Figure 3C, Figure 4B left, Table 2, Table 3, or the Fisher's test for Figure 4B right. Pearson correlation was calculated in Figure 1D. Statistics for Table 1 and Table 4 were specified in the footnotes. Hierarchical clustering in Figure 1C and Figure 3D was performed with the web tool ClustVis.^22^ Pathway enrichment analysis in Figure 3A was performed with the web server g:Profiler.^23^ The web server miRNeT was used for miRNA‐centered network analysis in Figure 3B.^24^

**TABLE 2:** Candidate targets of miR‐155 and miR‐200 in gastric diffuse large B‐cell lymphomas resistant (R) or sensitive (S) to *H. pylori* eradication therapy

Candidate targets of miR‐155					Candidate targets of miR‐200				
Gene	R	S	R/S	*p*	Gene	S	R	S/R	*p*
*DEPTOR*	555	1613	0.34	0.005	*LGALS9C*	35,845	13,308	2.69	0.0004
*EGLN3*	651	818	0.8	0.016	*LGALS9*	3237	1501	2.15	0.006
*SCNN1A*	283	835	0.34	0.019	*SAMD12*	426	169	2.5	0.008
*FCGR2A*	3263	474	6.88	0.036	*SELL*	303	2676	0.11	0.04
*ACVR1C*	142	175	0.81	0.12	*CXCL10*	289	1414	0.2	0.09
*COPZ2*	156	129	1.21	0.13	*CXCL9*	1656	6578	0.25	0.09
*DPP9*	662	519	1.28	0.13	*IFITM3*	12,370	28,154	0.44	0.10
*KRTAP5‐3*	62	53	1.16	0.15	*CCL4*	1929	3818	0.51	0.12
*KIF26A*	67	52	1.29	0.23	*IFITM1*	2226	4952	0.45	0.12
*F8*	1203	1433	0.84	0.27	*REXO1L1*	67	91	0.74	0.12
*NUDT10*	1377	1486	0.93	0.27	*AICDA*	38	585	0.064	0.15
*TAS2R20*	8	14	0.62	0.27	*C1QB*	2891	6444	0.45	0.16
*AQP6*	109	126	0.87	0.32	*TMPRSS4*	853	633	1.34	0.19
*GRK1*	195	220	0.89	0.37	*AIRE*	1061	900	1.18	0.22
*WDR7*	717	584	1.23	0.38	*PTGS1*	292	219	1.33	0.23
*ICA1L*	17	21	0.83	0.43	*AIM2*	1335	2990	0.45	0.28
*CYP21A2*	1042	1099	0.95	0.65	*ZNF431*	10	15	0.72	0.28
*PIGK*	260	236	1.1	0.68	*CCDC24*	42	58	0.72	0.29
*ABI3BP*	567	597	0.95	0.75	*CEBPA*	446	350	1.28	0.3
*RBPMS*	526	546	0.96	0.82	*GTF2IRD2B*	266	208	1.28	0.36
*KCNC1*	69	72	0.96	0.89	*CACNA1B*	1175	1105	1.06	0.45
					*LY6E*	522	642	0.81	0.53
					*NRXN2*	184	194	0.94	0.72
					*ST3GAL4*	357	447	0.8	0.72
					*EME2*	177	183	0.97	0.76
					*SLCO4A1*	233	215	1.08	0.85
					*COL27A1*	3849	3915	0.98	0.86
					*STRA8*	8.6	8.4	1.02	0.91
					*IFI44L*	620	602	1.03	0.92
					*B3GNT7*	492	345	1.42	1.43

Transfection of miR‐155 into BJAB cells identified 22 suppressed transcripts (Table S1). Levels of the candidate targets in biopsy specimens of gastric DLBCLs are listed here. Only *DEPTOR* was significantly reduced in *HPET*‐resistant gastric DLBCLs.

Transfection of miR‐200 into U2932 cells identified 30 suppressed transcripts. (Table S2). Levels of the candidate targets in biopsy specimens of gastric DLBCLs are listed here. Only *SELL* was significantly reduced in *HPET*‐sensitive gastric DLBCLs.

Abbreviations: DLBCL, diffuse large B‐cell lymphoma; HPET, *H. pylori* eradication therapy.

**TABLE 3:** miR‐155‐centered immune response genes

Gene	R*	S*	*p*	Description
*IFI27*	24,288	74,040	0.01	Interferon, alpha‐inducible protein 27 [NM_005532]
*NUPL2*	23,926	16,295	0.01	Nucleoporin like 2 [ENST00000477844]
*TLR5*	8531	22,005	0.01	Toll‐like receptor 5 [NM_003268]
*INPP5D*	6121	1641	0.03	Inositol polyphosphate 5‐phosphatase, 145 kDa, [NM_001017915]
*CD81*	3943	1752	0.01	CD81 molecule [NM_004356]
*ICAM3*	3720	1625	<0.01	Intercellular adhesion molecule 3 [NM_002162]
*LGALS9*	1502	3237	0.01	Lectin, galactoside‐binding, soluble, 9 [ENST00000584386]
*ERBB2*	1203	2528	0.01	V‐erb‐b2 avian erythroblastic leukemia viral oncogene homolog 2 [NM_001005862]
*ICAM1*	1888	586	0.02	Intercellular adhesion molecule 1 [NM_000201]
*WASL*	593	1378	<0.01	Wiskott‐Aldrich syndrome like [NM_003941]
*CDH1*	367	1529	0.01	Cadherin 1, type 1, E‐cadherin (epithelial) [NM_004360]
*JAK3*	1073	526	<0.01	Janus kinase 3 [NM_000215]
*PRKAR1A*	781	459	0.01	Protein kinase, cAMP‐dependent, regulatory, type I, alpha, [NM_212472]
*MAP3K14*	665	393	0.03	Mitogen‐activated protein kinase kinase kinase 14 [NM_003954]
*CBL*	527	386	0.02	Cbl proto‐oncogene, E3 ubiquitin protein ligase [NM_005188]
*STAT3*	596	254	0.01	Signal transducer and activator of transcription 3 (acute phase response factor) [NM_213662]
*PIK3CD*	555	267	0.01	Phosphatidylinositol 4,5‐bisphosphate 3‐kinase, catalytic subunit delta [NM_005026]
*KPNA7*	161	611	<0.01	Karyopherin alpha 7 (importin alpha 8) [NM_001145715]
*DOCK1*	328	405	0.02	Dedicator of cytokinesis 1 [NM_001380]
*CXADR*	121	482	<0.01	Coxsackie virus and adenovirus receptor [NM_001338]
*TLR3*	140	419	0.01	Toll‐like receptor 3 [NM_003265]
*BTC*	124	417	<0.01	Betacellulin [NM_001729]
*STAT5A*	396	129	0.01	Signal transducer and activator of transcription 5A [NM_003152]
*MAP3K13*	145	265	0.02	Mitogen‐activated protein kinase kinase kinase 13, [NM_004721]
*ERBB3*	84	294	<0.01	V‐erb‐b2 avian erythroblastic leukemia viral oncogene homolog 3, [NM_001005915]
*HAVCR2*	272	66	0.01	Hepatitis A virus cellular receptor 2 [NM_032782]
*PRKCB*	235	83	0.01	Protein kinase C, beta, [NM_002738]
*CNPY3*	202	93	<0.01	Canopy FGF signaling regulator 3 [NM_006586]
*TRIM32*	98	68	0.04	Tripartite motif‐containing 32 [NM_012210]
*CTLA4*	111	33	0.04	Cytotoxic T‐lymphocyte‐associated protein 4 [NM_005214]
*FGF7*	85	42	0.05	Fibroblast growth factor 7[NM_002009]
*NOD1*	62	25	<0.01	Nucleotide‐binding oligomerization domain‐containing 1 [NM_006092]

Abbreviation: HPET: *H. pylori* eradication therapy.

* The Table lists 32 genes that are differentially expressed between HPET‐resistant (R) and HPET‐sensitive (S) gastric diffuse large B‐cell lymphomas. Genes with high expression levels are at the top.

**TABLE 4:** Distinct pathology of early stage gastric diffuse large B‐cell lymphomas sensitive or resistant to *H. pylori* eradication therapy

	HPET sensitive	HPET resistant	*p* *
Gastritis			
Active	6	2	0.04
Mixed	7	3	
Chronic	3	9	
qRT‐PCR			
dCT _U6‐miR‐155_	−10.4 ± 0.2, *n* = 15	−8.6 ± 0.8, *n* = 13	0.02
dCT _ACTB‐DEPTOR_	0.9 ± 0.6, *n* = 17	−2.1 ± 0.7, *n* = 16	0.004
dCT _ACTB‐TLR5_	−1.2 ± 0.6, *n* = 17	−4.0 ± 0.5, *n* = 16	0.001
IHC			
%, pS6K1+ cells	33%, *n* = 15	56%, *n* = 15	0.005

Abbreviation: HPET: *H. pylori* eradication therapy.

* The Fisher's test for categorical data and the Student's *t*‐test for continuous data.

## 3 RESULTS

### 3.1 In gastric DLBCLs treated with *H. pylori* eradication therapy, the resistant group had higher levels of miR‐155 but lower levels of miR‐200 than the sensitive group

Using the NanoString technology, we measured genome‐wide miRNA expression profiles in nine *H. pylori* eradication therapy‐sensitive gastric DLBCLs and eight *H. pylori* eradication therapy‐resistant gastric DLBCLs. Out of 654 miRNAs, 2 miRNAs, including miR‐155 and miR‐1203, were associated with the resistant group, whereas 8 miRNAs, including miR‐200 a, b, and c, 22, 31, 141, 192, and 194, were associated with the sensitive group (Figure 1A; Data S1).

A two‐dimensional scatter plot of the expression levels of the 654 miRNAs showed that most miRNAs had similar levels in the two groups and were located near the diagonal (Figure 1B). There was more random scattering of miRNAs of low abundance at the left lower part of the scatter plot. Therefore, the more abundant miR‐155 and miR‐200 a, b, and c that were located off the diagonal, were chosen for further investigation.

Hierarchical clustering confirmed a resistant cluster including eight resistant cases and one sensitive case, and a sensitive cluster including eight sensitive cases (Figure 1C). The resistant cluster was associated with high levels of miR‐155, but low levels of miR‐200, miR‐192, and miR‐194. Conversely, the sensitive cluster was associated with low levels of miR‐155, but high levels of miR‐200, miR‐192, and miR‐194.

In addition, there was an inverse correlation between miR‐155 and miR‐200 a, b, or c. The correlation coefficients were −0.53, −0.63, and −0.58 at a *p* value of 0.12, 0.008, and 0.03, respectively, in the 17 gastric DLBCLs (Figure 1D). This finding suggested miR‐155 as a marker for resistance to *H. pylori* eradication therapy and miR‐200 as a marker for sensitivity to *H. pylori* eradication therapy.

### 3.2 mRNA profiles of miR‐155‐transfected BJAB cells and miR‐200‐transfected U2932 cells identified candidate targets of miR‐155 and miR‐200

BJAB and U2932 cells were derived from B‐cell lymphomas. To search for targets of miR‐155 or miR‐200, genome‐wide mRNA profiling was performed in BJAB cells transfected with miR‐155 and in U2932 cells transfected with miR‐200 a, b, or c. Genome‐wide alterations in mRNA profiles induced by miR‐155 and miR‐200 were obtained (Data S2 and S3).

B‐cell lymphomas had 10‐ to 30‐fold higher expression levels of miR‐155 than circulating B cells.^25^ We measured dCT_U6‐miR‐155_ in cell lines of diffuse large B‐cell lymphoma (−7.6 for BJAB, −7.7 for U2932, −8.2 for OCI‐Ly3, and −10.0 for OCI‐Ly7), Burkitt lymphoma (−9.9 for EBV‐negative Ramos and −10.7 for EBV‐positive Raj), and Hodgkin lymphoma (−4.5 for L428 and −6.2 for KHM2). After transfection of miR‐155 into BJAB cells, there was an increase in dCT_U6‐miR‐155_ from −7.6 to −5.3, indicating a fourfold increase in miR‐155, similar to the fourfold increase in miR‐155 from the sensitive group to the resistant group. For identification of miR‐155 targets, the absolute level of miR‐155 was probably not as critical as the changes induced by transfection.

The genes whose transcripts were suppressed by miR‐155 in BJAB cells or by miR‐200 in U2932 cells, including 22 candidate targets for miR‐155 and 30 candidate targets for miR‐200, are summarized in Table S1 and S2.

### 3.3 mRNA profiles of biopsy specimens verified that miR‐155 inhibited DEPTOR in gastric DLBCLs resistant to *H. pylori* eradication therapy

Genome‐wide mRNA profiles in eight resistant gastric DLBCLs and eight sensitive gastric DLBCLs were obtained (Data S4). The levels of miR‐155 or miR‐200 candidate targets in gastric DLBCLs are listed in Table 2. Out of the 22 candidate targets for miR‐155, DEPTOR was the one most significantly reduced in resistant gastric DLBCLs (*p *= 0.005). Out of the 30 candidate targets for miR‐200, only SELL was marginally reduced in sensitive gastric DLBCLs (*p* = 0.04).

Significantly, DEPTOR is a predicted target of miR‐155, but SELL is not a predicted target of miR‐200 a, b, or c, by several online programs, such as RNAhyb or the program at microRNA.org. Therefore, combined data from cell lines, gastric DLBCLs, and predictive programs implied that miR‐155 inhibits DEPTOR in gastric DLBCLs resistant to *H. pylori* eradication therapy.

### 3.4 Activation of mTOR through miR‐155‐mediated inhibition of DEPTOR in lymphoma cell lines

Experiments were performed in BJAB and U2932 cells to confirm that mTOR could be activated by miR‐155‐mediated inhibition of DEPTOR.

**FIGURE 2:**
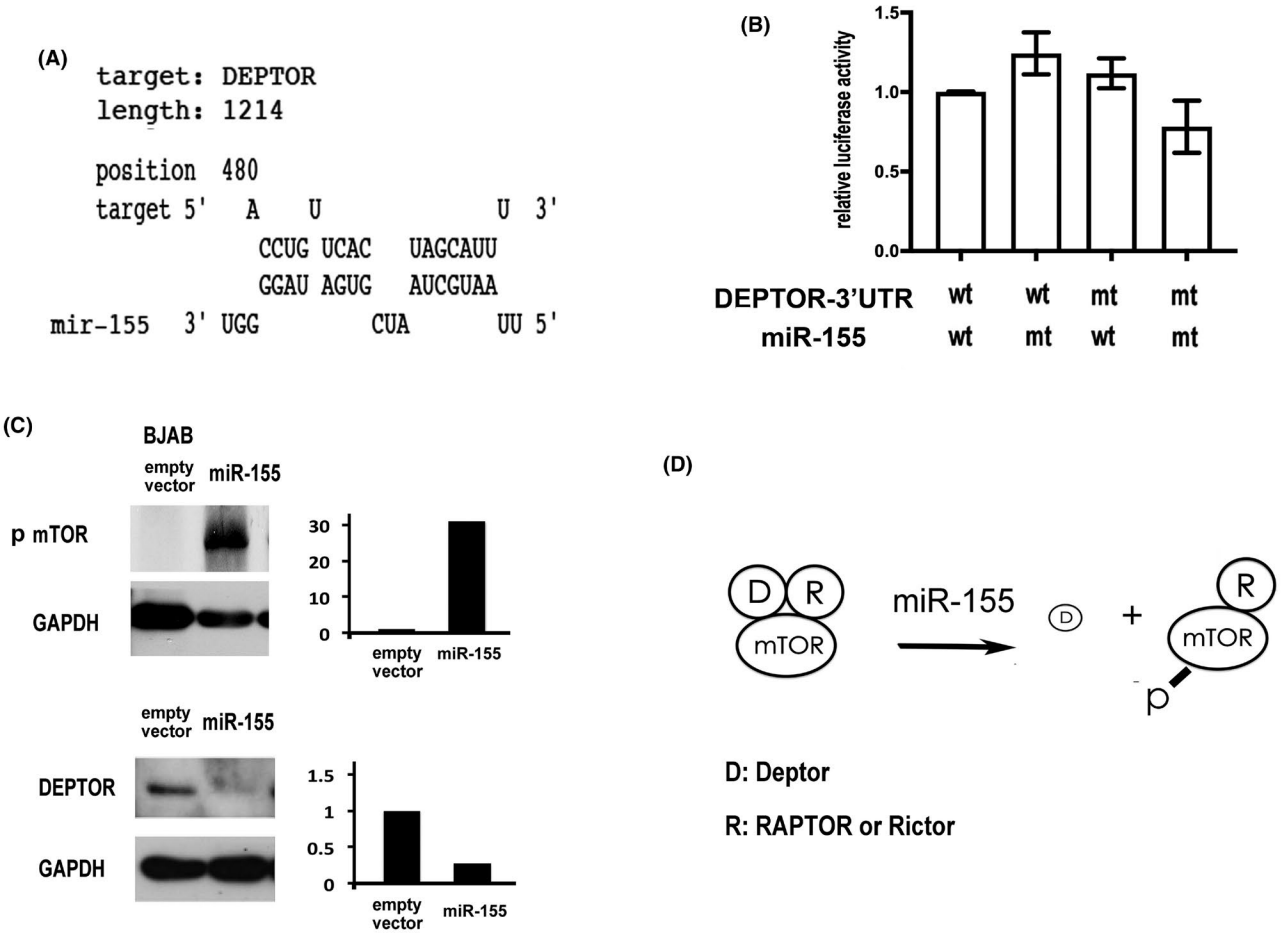
MiR‐155 inhibits DEPTOR with secondary activation of mTOR in B lymphoma cell lines. (A) A model for the interactions between miR‐155 and the 3'UTR of DEPTOR. The RNAhyb program predicts a binding site starting at 480 nucleotides after the stop codon. Upper: sequence of the target site at the 3'UTR of DEPTOR; Lower: sequence of miR‐155. The 5' and 3' ends are specified. (B) Confirmation of the binding site for miR‐155 with luciferase assay in U2932 cells. Y‐axis: relative luciferase activity. X‐axis, from left to right: U2932 cells transfected with miR‐155 and DEPTOR, with mt‐miR‐155 and DEPTOR, with miR‐155 and mt DEPTOR, and with mt miR‐155 and mt DEPTOR (wt: wild type; mt mutant). (C) Induction of phosphorylated mTOR (p‐mTOR) by miR‐155 in BJAB cells. An EGFP+ vector is used to over‐express miR‐155. After transfection and sorting, >90% of the cells are EGFP+. Overexpression of miR‐155 reduced the level of DEPTOR with secondary increase in the level of p‐mTOR. (D) A model for induction of p‐mTOR by miR‐155 in gastric diffuse large B‐cell lymphoma resistant *to H. pylori* eradication therapy. In the mTOR complex, DEPTOR together with either RAPTOR in mTORC1 or RICTOR in mTORC2 inhibit the activity of mTOR. In the presence of increased miR‐155 and decreased DEPTOR, the inactive complex dissociates into active form. D: DEPTOR and R: RAPTOR or RICTOR

The interactions between miR‐155 and the 3'UTR of DEPTOR, based on the RNAhyb program, were located at 480 nucleotides after the stop codon (Figure 2A). Confirmation of the binding site for miR‐155 was performed with luciferase assays in U2932 cells (Figure 2B). The activity of luciferase was suppressed when miR‐155 interacted with 3'UTR of DEPTOR. Disruptions of the interactions, due to mutations of either miR‐155 or 3'UTR of DEPTOR, increased the activity of luciferase. Restorations of the interactions, after simultaneous compensatory mutations of both miR‐155 and 3'UTR of DEPTOR, suppressed the luciferase activity again. These compensatory mutations confirmed the predicted interactions in Figure 2A.

Furthermore, western blotting showed that overexpression of miR‐155 in BJAB cells inhibited DEPTOR and induced phosphorylated mTOR (p‐mTOR) (Figure 2C). Together, we proposed a model for resistance to *H. pylori* eradication, in which miR‐155 may inhibit DEPTOR, leading to dissociation of the inactive mTOR complex and activation of p‐mTOR (Figure 2D).

### 3.5 Pathway enrichment analysis linked immune response pathways with responses to *H. pylori* eradication therapy

**FIGURE 3:**
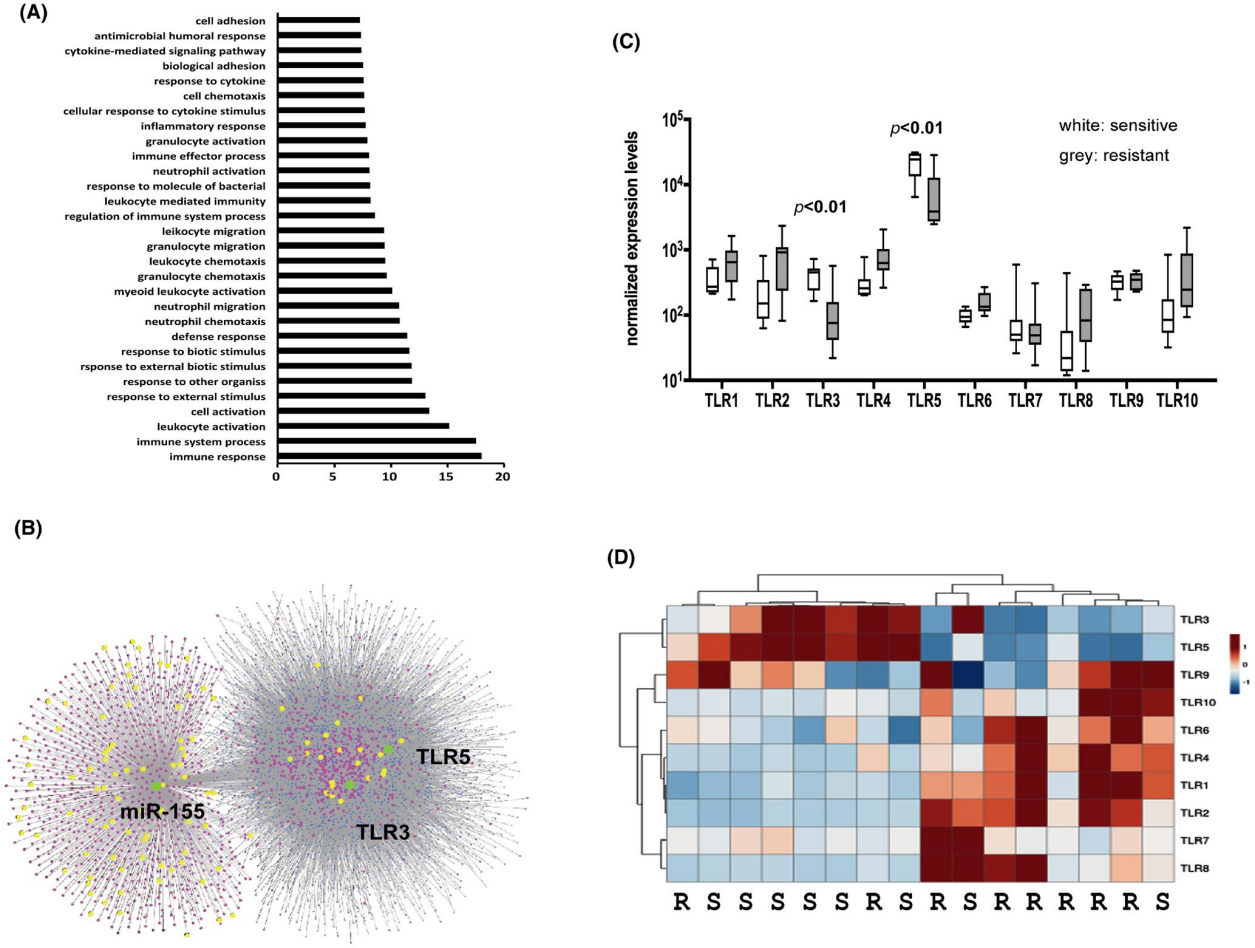
A miR‐155‐related immune network identified Toll‐like receptor 5 as a potential marker for sensitivity to *H. pylori* eradication therapy. (A) Pathway enrichment analysis linked immune response pathways with responses to *H. pylori* eradication therapy. Genome‐wide mRNA expression profiles in eight sensitive and eight resistant gastric DSLBCLs identified 563 differentially expressed mRNAs. The online program g:Profiler is used for pathway enrichment analysis on the Gene Ontology (GO) database. The immune response pathway is the most significantly enriched GO pathway. X‐axis: *p* value for enrichment, adjusted, in –log scale; Y‐axis: significantly enriched pathways, with the most significant one at the bottom. (B) TLR5 and TLR3 in a miR‐155‐centered immune network. An immune network is constructed, using miR‐155 and 563 differentially expressed mRNAs as inputs into the online program miRNet. The constructed network included miR‐155 (green), intermediate miRNAs (blue), 73 non‐differentially expressed mRNAs (red), and 32 differentially expressed mRNAs (yellow). The left cluster is due to mRNAs (red & yellow) regulated by miR‐155 (green) and the right cluster is due to mRNAs (red & yellow) indirectly connected with miR‐155 via intermediate miRNAs (blue). (C) The expression levels of TLR*s* 1–10 in gastric diffuse large B‐cell lymphomas: TLR5 is a potential predictor of sensitivity to *H. pylori* eradication therapy. The expression levels of TLRs are extracted from genome‐wide mRNA profiles of eight sensitive and eight resistant cases. The sensitive group had higher expressions of both TLR3 and TLR5 than the resistant group (*p* < 0.01). Note that the expression level of TLR5 is more than one log higher than all the other TLRs. (D) Hierarchical clustering of gastric diffuse large B‐cell lymphomas with TLRs 1–10. The left sensitive cluster included six sensitive and two resistant cases associated with higher expression levels of TLR3 and TLR5. In contrast, the right resistant cluster included two sensitive and six resistant cases associated with higher expression levels of the remaining eight TLRs. R: resistant and S: sensitive

Genome‐wide mRNA expression profiles in eight *H. pylori* eradication therapy‐sensitive gastric DLBCLs and eight *H. pylori* eradication therapy‐resistant gastric DLBCLs identified 563 mRNAs that were differentially expressed in the sensitive versus resistant group. The online program g:Profiler was used for pathway enrichment analysis based on the Gene Ontology (GO) database. The 30 most enriched pathways are shown in Figure 3A, with the immune response pathway being the most significantly enriched GO pathway.

### 3.6 Construction of a miR‐155‐centered immune network including TLR5

Using miR‐155 and the 563 mRNAs as inputs into the online program miRNet, a miR‐155‐centered immune network was constructed, including 73 non‐differentially expressed mRNAs and 32 out of the 563 differentially expressed mRNAs (Figure 3B). The network included a cluster of mRNAs directly inhibited by miR‐155 and a second cluster of mRNAs indirectly connected with miR‐155. TLR5 and also TLR3, belonged to the indirect cluster (Figure 3B).

The expression levels of these 32 differentially expressed mRNAs are listed in Table 3. TLR5, one of the genes with high expression levels, was associated with sensitivity to *H. pylori* eradication therapy. Another Toll‐like receptor, TLR3, was also associated with sensitivity *to H. pylori* eradication therapy, although the expression level of TLR3 was much lower than that of TLR5 (TLR5: 8531 in the resistant group and 22,005 in the sensitive group; TLR3: 140 in the resistant group and 419 in the sensitive group).

It is interesting to know that TLR5 is a cell membrane receptor for bacterial flagellin, such as that of *H. pylori*, and TLR3 is an intracellular receptor for double‐stranded RNA of viral origin.

### 3.7 The expression levels of TLRs 1–10 in gastric DLBCLs: TLR5 was a potential predictor of sensitivity to *H. pylori* eradication therapy

The expression levels of TLRs were extracted from genome‐wide mRNA profiles of eight *H. pylori* eradication therapy‐sensitive gastric DLBCLs and eight *H. pylori* eradication therapy‐resistant gastric DLBCLs (Figure 3C). The sensitive group had higher expressions of both TLR3 and TLR5 than the resistant group (*p* < 0.01). In addition, the expression level of TLR5 was more than one log higher than all the other TLRs, implying a critical role of TLR5 in predicting sensitivity to *H. pylori* eradication therapy.

### 3.8 Hierarchical clustering of gastric DLBCLs with TLRs 1–10: association of TLR5 with *s*ensitivity to *H. pylori* eradication therapy

The expression levels of all 10 TLRs in 8 *H. pylori* eradication therapy‐sensitive gastric DLBCLs and 8 *H. pylori* eradication therapy‐resistant gastric DLBCLs were used in hierarchical clustering (Figure 3D). Higher levels of TLR3 and TLR5 were associated with a cluster of six sensitive and two resistant lymphomas. In contrast, higher expression levels of the remaining eight TLRs were associated with a cluster of two sensitive and six resistant lymphomas. This observation provided further evidence for using TLR5 as a marker of sensitivity to *H. pylori* eradication therapy in gastric DLBCLs.

### 3.9 In gastric DLBCLs treated with *H. pylori* eradication therap*y*, sensitivity was correlated with active gastritis and weaker pS6K1, whereas resistance was correlated with chronic gastritis and stronger pS6K1

Because TLR5 increases neutrophil infiltration in animal models of bacterial infection,^26^, ^27^ histopathological evidences of acute inflammation could be used as surrogate markers for sensitivity to *H. pylori* eradication therapy. Conversely, activation of mTOR causes fibrosis in several organ systems,^28^, ^29^, ^30^, ^31^, ^32^ so chronic inflammation or increase in the mTOR‐regulated phosphorylation of S6K1 might be used as surrogate markers for resistance to *H. pylori* eradication therapy.

**FIGURE 4:**
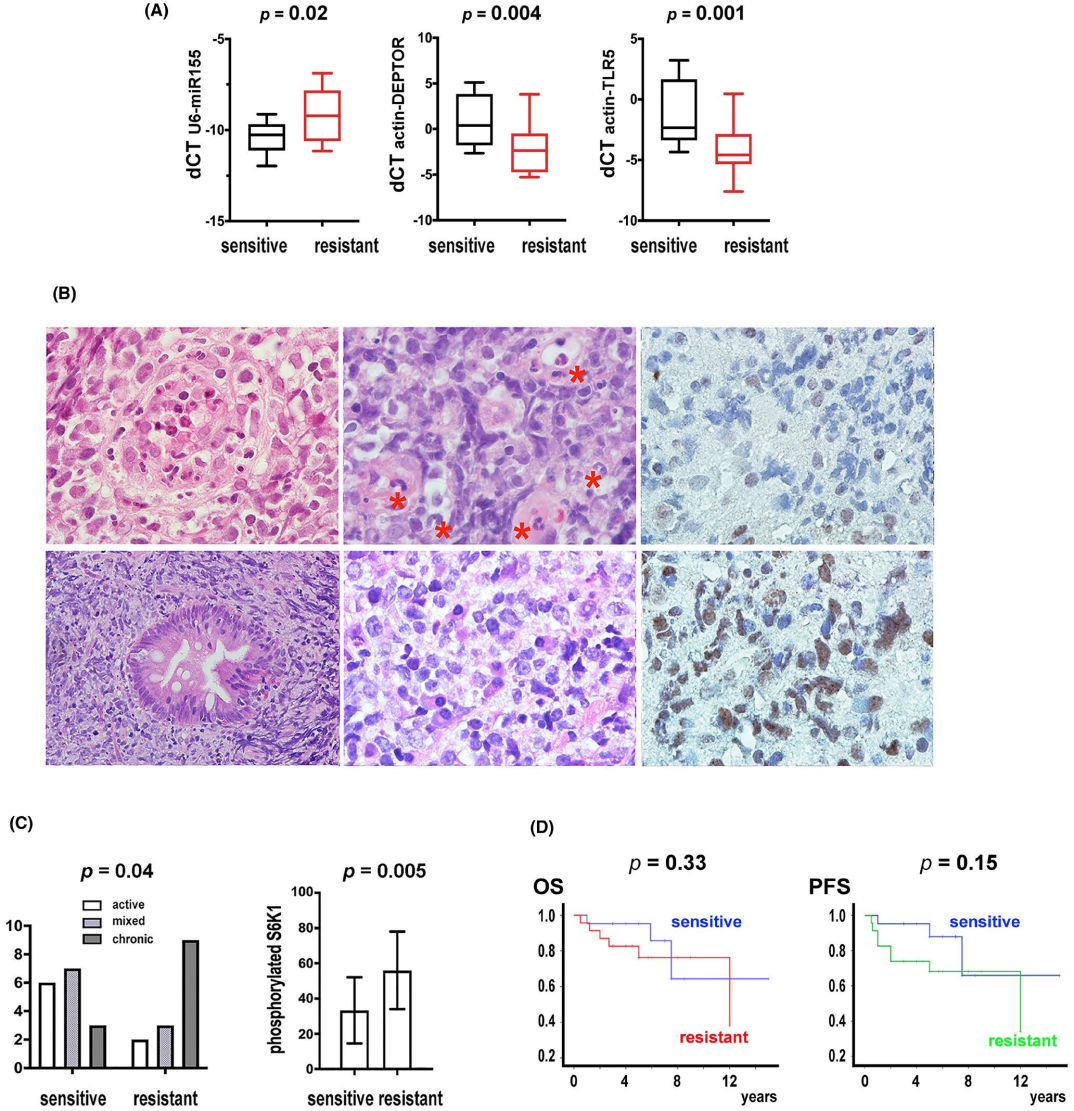
Sensitivity to *H. pylori* eradication therapy is correlated with active gastritis; resistance to *H. pylori* eradication therapy is correlated with phosphorylated S6K1. (A) The resistant group had higher levels of miR‐155 and lower levels of DEPTOR and TLR5 than the sensitive group. From left to right: X‐axis: sensitive and resistant groups; Y‐axis: dCT of U6‐miR‐155, actin‐DEPTOR, and actin‐TLR5. (B) Representative histopathology of active and chronic gastritis and immunohistochemistry of pS6K1 in gastric diffuse large B‐cell lymphomas. Upper: lymphoma cells surrounding a destroyed foveolar gland with intraluminal neutrophils and debris (left), lymphoma cells in a vascular‐rich stroma with intravascular accumulation of neutrophils (middle), and weak pS6K1 expression (right). Lower: crushed lymphoma cells in a fibrotic stroma and a foveolar gland with intestinal metaplasia (left) and interstitial plasmacytosis (middle), and strong pS6K1 expression on lymphoma cells (right). H/E and immunoperoxidase stains on formalin‐fixed paraffin‐embedded tissue sections (all 1000×, except for the lower left at 400×). (C) The sensitive group is associated with active gastritis; the resistant group is associated with the expression of phosphorylated S6K1. Left: X‐axis: sensitive and resistant groups; Y‐axis: case numbers with active, mixed, chronic gastritis. Right: X‐axis: sensitive and resistant groups; Y‐axis: percentages of phosphorylated S6K1+ cells by immunohistochemistry, mean ± SE. (D) Overall survival (OS) and progression‐free survival (PFS) for the sensitive group are similar to that of the resistant group secondarily treated with chemotherapy. The 5‐year OS is 95.2% (95% CI, 90.6%–99.8%) for 21 sensitive cases and 76.3% (95% CI, 57.7%–97.9%) for 23 resistant cases that also received chemotherapy after failure to *H. pylori* eradication therapy (*p* = 0.33). The 5‐year PFS is 95.2% (95% CI, 90.6%–99.8%) for the sensitive group and 68.2% (95% CI, 48.6%–87.8%) for the resistant group (*p *= 0.15)

A series of early stage gastric DLBCLs that has been treated with *H. pylori* eradication therapy was used to support this hypothesis. The clinical and pathological features are summarized in Table 1 and Table 4. The sensitive group was characterized by lower miR‐155 and higher DEPTOR and TLR5 than the resistant group (Figure 4A).

Active inflammation was characterized by foveolar abscess of the gland, a vascular‐rich stroma with intravascular and interstitial neutrophilic infiltrates, and weak pS6K1 expression (Figure 4B, upper). Chronic inflammation was characterized by intestinal metaplasia of the gland, a fibrotic stroma with plasmacytoid infiltrates, and strong pS6K1 expression (Figure 4B, lower).

The sensitive group was more likely to have active inflammation and the resistant group was more likely to have chronic inflammation (*p *= 0.04, Figure 4C, left). The sensitive group was more likely to have weaker pS6K1 and the resistant group was more likely to have stronger pS6K1 (*p* = 0.005, Figure 4C, right).

### 3.10 Patients who were resistant to HPET may be successfully rescued by secondary immunochemotherapy without any detrimental effects

The sensitive group included 21 patients that achieved complete remission after *H. pylori* eradication therapy (HPET). The median time to complete remission was 2 months. None had later relapse. The 5‐year overall survival (OS) or progression‐free survival (PFS) was 95.2% (95% CI, 90.6%–99.8%), and there was no need for second‐line treatment.

The resistant group included 23 patients with residual, stable, or progressive disease after HPET. They were then treated with chemotherapy. The 5‐year OS was 76.3% (95% CI, 57.7%–97.9%), and the 5‐year PFS was 68.2% (95% CI, 48.6%–87.8%). The median TTNT (time to next treatment) from initiation of HPET to initiation of chemotherapy was 4 months.

In this series, 5 of the 21 sensitive cases and 3 of the 23 resistant cases received more than one course of HPET (*p *= 0.45, Fisher's test). The differences in the OS or PFS between the sensitive and the resistant groups were not significant (*p* = 0.33 for OS, and *p* = 0.15 for PFS, Figure 4D). HPET for the sensitive group was as effective as secondary chemotherapy for the resistant group, but without the complications of chemotherapy.

## 4 DISCUSSION

Gastric lymphoma, including MALT lymphoma and DLBCL, is often associated with *H. pylori* infection. Both MALT lymphoma and DLBCL can be treated by *H. pylori* eradication therapy.^2^, ^3^, ^4^ Depending on responses to *H. pylori* eradication therapy, gastric DLBCLs include a sensitive group and a resistant group. However, the molecular features that distinguish the sensitive group from the resistant group are still unclear. Through miRNA profiles, we showed miR‐155 as a marker for resistance to *H. pylori* eradication therapy. To identify the miR‐155 target, we transfected miR‐155 into BJAB cells to isolate a list of 22 candidate targets, and showed that DEPTOR was the only target suppressed in the resistant group. This is an experimental approach. In contrast, the identification of TLR5 as a marker for the sensitive group was performed with the miRNet, which is a recently established web server for miRNA and mRNA interactions.^24^ These complementary approaches then suggested active gastritis, which correlates with TLR5, and phosphorylated S6K1, a substrate of mTOR, as the most practical surrogate markers for responses to *H. pylori* eradication therapy.

The protein mTOR is normally in complex with an inhibitory regulator, either RAPTOR in mTOR complex 1 (mTORC1) or RICTOR in mTOR complex 2 (mTORC2). On top of these inhibitory regulators, DEPTOR inhibits mTOR through direct interactions with mTORC1 or mTORC2 (Figure 2D). Both RAPTOR and RICTOR are known targets of miR‐155.^7^, ^33^ And in the present report, we further demonstrated that miR‐155 may inhibit DEPTOR with activation of mTOR to cause resistance to *H. pylori* eradication therapy in gastric DLBCL. This conclusion extends a previous report that miR‐155 is a biomarker of resistance to *H. pylori* eradication therapy in gastric MALT lymphoma.^34^

The mTOR pathway is not only involved in cell metabolism and cell proliferation, as initially thought, but also related to immunity against bacterial and viral infection,^35^, ^36^, ^37^ including *H. pylori* infection.^38^ Due to the complexity of T cells and dendritic cells involved in immune reactions, mTOR activation may either protect the host or promote the disease.

Similar to the mTOR pathway, miR‐155 is an evolutionarily ancient miRNA that not only regulates the immune system,^39^, ^40^, ^41^, ^42^ with significant roles in bacterial infection,^43^ but also has been used as a biomarker for disease progression in B‐cell lymphoma.^44^ Consistent with these data on miR‐155 and mTOR, we identified DEPTOR as a novel target of miR‐155, and established associations between the miR‐155‐DEPTOR‐mTOR pathway and resistance to *H. pylori* eradication therapy in gastric DLBCLs. DEPTOR, a target of microRNA‐155, regulates migration and cytokine production in nodal diffuse large B‐cell lymphoma.^45^ Therefore, the association between suppression of DEPTOR and a more aggressive behavior is not specific for gastric DLBCL. However, the biological mechanisms and clinical significance in gastric DLBCL, due to resistance to eradication therapy through the mTOR pathway, are different from those of nodal DLBCL, due to cytokine production.

TLR5 recognizes flagellin of various bacteria, and increases neutrophil infiltration in animal models of bacterial infection.^26^, ^27^ Although *H. pylori* have evolved mutant flagellin with low affinity for TLR5 to evade the immune system of the host, the *H. pylori* encoded protein cagL or cagY can still act as a flagellin‐independent TLR5 activator, and expression of TLR5 was correlated with active gastritis in murine models of *H. pylori* infection.^46^, ^47^ These observations are consistent with our findings that active gastritis is associated with strong TLR5 expression and sensitivity to *H. pylori* eradication therapy in gastric DLBCLs. However, due to the small sample size, mixed acute and chronic inflammation, and variability in morphological assessment, an independent series would be necessary to confirm the usage of active gastritis as a marker for sensitivity to *H. pylori* eradication therapy.

TLR5 probably did not upregulate miR‐155, because the correlation coefficient between dCT_U6‐miR‐155_ and dCT_actin‐TLR5_ was −0.22, at *p* value of 0.35, in our series. It is interesting to note that the promoter of miR‐155 includes two NFκB binding sites,^48^ and *H. pylori* may activate NFκB via the alternative pathway in B lymphocytes.^49^ Therefore^,^ *H. pylori* might have induced miR‐155 through NFκB in gastric DLBCL, and the elevated miR‐155 might cause resistance to *H. pylori* eradication therapy.

Initially, we thought that the GCB versus ABC classification might be associated with responses to *H. pylori* eradication therapy. BJAB is a cell line of GCB (germinal center B cell) DLBCL, and miR‐155 was transfected into BJAB cells to search for inhibition of GCB‐related mRNAs. Similarly, U2932 is a cell line of ABC (activated B cell) DLBCL, and miR‐200 was transfected into U2932 to search for inhibition of ABC‐related mRNAs. Our negative finding of miR‐155 or miR‐200‐induced inhibition of GCB or ABC‐associated transcripts suggested that the division into GCB and ABC types of gastric DLBCL might not be as significant as that of nodal DLBCL.

Because of an association between reduced DEPTOR and an aggressive behavior in nodal DLBCL of the ABC type,^45^ we have looked for a similar association in gastric DLBCL through the GCB versus ABC classification. However, the classification of gastric DLBCL into GCB and ABC types is itself an issue. We have tried clustering of our gastric DLBCLs into GCB and ABC types, using as controls, NCBI GEO GSE56315.^50^ Out of 16 cases from our series with genome‐wide expression profiles, only 3 cases were classified as the GCB type and 3 cases were classified as the ABC type. The remaining 10 cases could not be classified due to mixed up of GCB and ABC markers (Figure S1). This result further demonstrated a difference between nodal and extra‐nodal diffuse large B‐cell lymphomas.

The prevalence of EBV infection was not investigated in our series. The diagnosis of EBV infection on biopsy is usually based on in situ hybridization for EBER. The prevalence of EBV infection in gastric lymphoma varies from rare to 20%, in part because no uniform cut‐off percentages of EBER+ cells were used. A recent meta‐analysis found an overall prevalence of about 7% for all nodal and non‐nodal DLBCLs.^51^ We did not look specifically at EBV infection, because 3 of our 44 cases might be EBV+, highly unlikely to draw a statistically significant conclusion on EBV and miR‐155 from such limited cases.

In conclusion, genome‐wide miRNA and mRNA profiling were performed on gastric diffuse large B‐cell lymphomas. In the first step, miR‐155 was identified as a marker for resistance to *H. pylori* eradication therapy. In the second step, DEPTOR was identified through an experimental approach as a novel target of miR‐155 and TRL5 was identified through a bioinformatic approach as a marker for sensitivity to *H. pylori* eradication therapy. In the third step, we showed active gastritis and phosphorylated S6K1 as surrogate markers for TLR5 and mTOR, respectively. Our data extend a previous report that miR‐155 is a biomarker of resistance to *H. pylori* eradication therapy in gastric MALT lymphomas.^34^ The activation of the miR‐155‐DEPTOR‐mTOR pathway is of biological and clinical significance in gastric DLBCL. Additional studies would be necessary to reveal how activation of mTOR induces resistance to *H. pylori* eradication therapy in gastric DLBCL.

## CONFLICT OF INTEREST

The authors have declared that no conflict of interest exists.

## AUTHOR CONTRIBUTION

Huang WT and Kuo YC performed the experiments. Kuo SH provided the clinical data and informed consents. Lin CW designed the project and wrote the manuscript.

## ETHICAL APPROVAL STATEMENT

The ethical committee of the National Taiwan University Hospital approved the study and informed consent was obtained.

## Supporting information

Fig S1

Table S1

Table S2

Data S1‐S4

## Data Availability

Genome‐wide expression profiles, including supplemental datasets 1, 2, 3, and 4, were deposited at NCBI GEO dataset as GSE182362. Plasmids would be made available upon request.

## References

[1] Swerdlow SH , Campo E , Pileri SA , et al. The 2016 revision of the World Health Organization classification of lymphoid neoplasms. Blood. 2016;127:2375‐2390. 26980727 10.1182/blood-2016-01-643569 PMC4874220

[2] Ferreri AJ , Montalban C . Primary diffuse large B‐cell lymphoma of the stomach. Crit Rev Oncol Hematol. 2007;63:65‐71. 17339119 10.1016/j.critrevonc.2007.01.003

[3] Psyrri A , Papageorgiou S , Economopoulos T . Primary extranodal lymphomas of stomach: clinical presentation, diagnostic pitfalls and management. Ann Oncol. 2008;19:1992‐1999. 18647965 10.1093/annonc/mdn525 PMC2733120

[4] Kuo S‐H , Yeh K‐H , Wu M‐S , et al. Helicobacter pylori eradication therapy is effective in the treatment of early‐stage H pylori‐positive gastric diffuse large B‐cell lymphomas. Blood. 2012;119:4838‐4844. 22403257 10.1182/blood-2012-01-404194

[5] Takei N , Nawa H . mTOR signaling and its roles in normal and abnormal brain development. Front Mol Neurosci. 2014;7:28. 24795562 10.3389/fnmol.2014.00028 PMC4005960

[6] Shimobayashi M , Hall MN . Making new contacts: the mTOR network in metabolism and signalling crosstalk. Nat Rev Mol Cell Biol. 2014;15:155‐162. 24556838 10.1038/nrm3757

[7] Huang X , Shen Y , Liu M , et al. Quantitative proteomics reveals that miR‐155 regulates the PI3K‐AKT pathway in diffuse large B‐cell lymphoma. Am J Pathol. 2012;181:26‐33. 22609116 10.1016/j.ajpath.2012.03.013 PMC3388146

[8] Chen H‐H , Huang W‐T , Yang L‐W , et al. The PTEN‐AKT‐mTOR/RICTOR pathway in nasal natural killer cell lymphoma is activated by miR‐494‐3p via PTEN but inhibited by miR‐142‐3p via RICTOR. Am J Pathol. 2015;185:1487‐1499. 25907832 10.1016/j.ajpath.2015.01.025

[9] Wang Z , Zhong J , Inuzuka H , et al. An evolving role for DEPTOR in tumor development and progression. Neoplasia. 2012;14:368‐375. 22745583 10.1593/neo.12542 PMC3384424

[10] Smith SM . Clinical development of mTOR inhibitors: a focus on lymphoma. Rev Recent Clin Trials. 2007;2:103‐110. 18473994 10.2174/157488707780599366

[11] Majchrzak A , Witkowska M , Smolewski P . Inhibition of the PI3K/Akt/mTOR signaling pathway in diffuse large B‐cell lymphoma: current knowledge and clinical significance. Molecules. 2014;19:14304‐14315. 25215588 10.3390/molecules190914304 PMC6271242

[12] Lee JS , Vo TT , Fruman DA . Targeting mTOR for the treatment of B cell malignancies. Br J Clin Pharmacol. 2016;82:1213‐1228. 26805380 10.1111/bcp.12888 PMC5061788

[13] Fitzgerald KA , Kagan JC . Toll‐like receptors and the control of immunity. Cell. 2020;180:1044‐1066. 32164908 10.1016/j.cell.2020.02.041 PMC9358771

[14] Nagashima H , Yamaoka Y , Nagashima H , et al. Importance of toll‐like receptors in pro‐inflammatory and anti‐inflammatory responses by helicobacter pylori infection. Curr Top Microbiol Immunol. 2019;421:139‐158. 31123888 10.1007/978-3-030-15138-6_6

[15] Isaza‐Correa JM , Liang Z , van den Berg A , et al. Toll‐like receptors in the pathogenesis of human B cell malignancies. J Hematol Oncol. 2014;7:57. 25112836 10.1186/s13045-014-0057-5 PMC4237867

[16] Meliț LE , Mărginean CO , Mărginean CD , et al. The Relationship between toll‐like receptors and helicobacter pylori‐related gastropathies: still a controversial topic. J Immunol Res. 2019;2019:8197048. 30863783 10.1155/2019/8197048 PMC6378784

[17] Uno K , Kato K , Shimosegawa T , et al. Novel role of toll‐like receptors in Helicobacter pylori ‐ induced gastric malignancy. World J Gastroenterol. 2014;20:5244‐5251. 24833854 10.3748/wjg.v20.i18.5244 PMC4017039

[18] Geiss GK , Bumgarner RE , Birditt B , et al. Direct multiplexed measurement of gene expression with color‐coded probe pairs. Nat Biotechnol. 2008;26:317‐325. 18278033 10.1038/nbt1385

[19] Malkov VA , Serikawa KA , Balantac N , et al. Multiplexed measurements of gene signatures in different analytes using the NanoString nCounter Assay System. BMC Res Notes. 2009;2:80. 19426535 10.1186/1756-0500-2-80 PMC2688518

[20] Kulkarni MM . Digital multiplexed gene expression analysis using the NanoString nCounter system. Curr Protoc Mol Biol. 2011;25:Unit25B.10. 10.1002/0471142727.mb25b10s94 21472696

[21] Magnuson B , Ekim B , Fingar DC . Regulation and function of ribosomal protein S6 kinase (S6K) within mTOR signalling networks. Biochem J. 2012;441:1‐21. 22168436 10.1042/BJ20110892

[22] Metsalu T , Vil J . Clustvis: a web tool for visualizing clustering of multivariate data using Principal Component Analysis and heatmap. Nucleic Acids Res. 2015;43:W566‐W570. 25969447 10.1093/nar/gkv468 PMC4489295

[23] Raudvere U , Kolberg L , Kuzmin I , et al. g:Profiler: a web server for functional enrichment analysis and conversions of gene lists (2019 update). Nucleic Acids Res. 2019;47:W191‐W198. 31066453 10.1093/nar/gkz369 PMC6602461

[24] Chang LE , Zhou G , Soufan O , et al. miRNet 2.0‐network‐based visual analytics for miRNA functional analysis and systems biology. Nucl. Acids Res. 2020;48:W244‐W251. 32484539 10.1093/nar/gkaa467 PMC7319552

[25] Eis PS , Tam S , Sun L , et al. Accumulation of miR‐155 and BIC RNA in human B cell lymphomas. Proc Natl Acad Sci USA. 2005;102:3627‐3632. 15738415 10.1073/pnas.0500613102 PMC552785

[26] Morris AE , Liggitt HD , Hawn TR , et al. Role of toll‐like receptor 5 in the innate immune response to acute P. aeruginosa pneumonia. Am J Physiol Lung Cell Mol Physiol. 2009;297:L1112‐L1119. 19801452 10.1152/ajplung.00155.2009 PMC2793188

[27] Parkunan SM , Astley R , Callegan MC . Role of TLR5 and flagella in bacillus intraocular infection. PLoS One. 2014;9(6):e100543. 24959742 10.1371/journal.pone.0100543 PMC4068998

[28] Lawrence J , Nho R , Lawrence J , et al. The role of the Mammalian Target of Rapamycin (mTOR) in pulmonary fibrosis. Int J Mol Sci. 2018;19:778. 10.3390/ijms19030778 PMC5877639 29518028

[29] Dany M , Rimmani HH , Matar SA , et al. mTORC2‐Akt signaling axis is implicated in myocardial compensation and fibrosis. J Biol Regul Homeost Agents. 2015;29:745‐753. 26753634

[30] Wei R , Liu H , Chen R , et al. Astragaloside IV combating liver cirrhosis through the PI3K/Akt/mTOR signaling pathway. Exp Ther Med. 2019;17:393‐397. 30651810 10.3892/etm.2018.6966 PMC6307369

[31] Geissler EK , Schlitt HJ , Geissler EK , et al. The potential benefits of rapamycin on renal function, tolerance, fibrosis, and malignancy following transplantation. Kidney Int. 2010;78(11):1075‐1079. 20861822 10.1038/ki.2010.324

[32] Ghadimi M , Dashti‐Khavidaki S , Khalili H , et al. mTOR inhibitors for management of encapsulating peritoneal sclerosis: a review of literatures. Ren Fail. 2016;38:1574‐1580. 27425661 10.1080/0886022X.2016.1209026

[33] Tsuchiya M , Kalurupalle S , Kumar P , et al. RPTOR, a novel target of miR‐155, elicits a fibrotic phenotype of cystic fibrosis lung epithelium by upregulating CTGF. RNA Biol. 2016;13:837‐847. 27284727 10.1080/15476286.2016.1197484 PMC5013987

[34] Saito Y , Suzuki H , Tsugawa H , et al. Overexpression of miR‐142‐5p and miR‐155 in gastric mucosa‐associated lymphoid tissue (MALT) lymphoma resistant to Helicobacter pylori eradication. PLoS One. 2012;7:e47396. 23209550 10.1371/journal.pone.0047396 PMC3509063

[35] Martin S , Saha B , Riley JL . The battle over mTOR: an emerging theatre in host‐pathogen immunity. PLoS Pathog. 2012;8:e1002894. 23028309 10.1371/journal.ppat.1002894 PMC3441621

[36] Abdel‐Nour M , Tsalikis J , Kleinman D , et al. The emerging role of mTOR signalling in antibacterial immunity. Immunol Cell Biol. 2014;92:346‐353. 24518980 10.1038/icb.2014.3

[37] Buchkovich NJ , Yu Y , Zampieri CA , et al. The TORrid affairs of viruses: effects of mammalian DNA viruses on the PI3K‐Akt‐mTOR signalling pathway. Nat Rev Microbiol. 2008;6:266‐275. 18311165 10.1038/nrmicro1855 PMC2597498

[38] Ko SH , da Rho J , Jeon JI , et al. Crude preparations of helicobacter pylori outer membrane vesicles induce upregulation of heme oxygenase‐1 via activating Akt‐Nrf2 and mTOR‐IkappaB Kinase‐NF‐kappaB pathways in dendritic cells. Infect Immun. 2016;84:2162‐2174. 27185786 10.1128/IAI.00190-16 PMC4962631

[39] Sokolova O , Vieth M , Gnad T , et al. Helicobacter pylori promotes eukaryotic protein translation by activating phosphatidylinositol 3 kinase/mTOR. Int J Biochem Cell Biol. 2014;55:157‐163. 25194338 10.1016/j.biocel.2014.08.023

[40] Vigorito E , Kohlhaas S , Lu D , et al. miR‐155: an ancient regulator of the immune system. Immunol Rev. 2013;253:146‐157. 23550644 10.1111/imr.12057

[41] Lind EF , Ohashi PS . Mir‐155, a central modulator of T‐cell responses. Eur J Immunol. 2014;44:11‐15. 24571026 10.1002/eji.201343962

[42] Montagner S , Orlandi EM , Merante S , et al. The role of miRNAs in mast cells and other innate immune cells. Immunol Rev. 2013;253:12‐24. 23550635 10.1111/imr.12042

[43] Staedel C , Darfeuille F . MicroRNAs and bacterial infection. Cell Microbiol. 2013;15:1496‐1507. 23795564 10.1111/cmi.12159

[44] Due H , Svendsen P , Bødker JS , et al. miR‐155 as a biomarker in B‐cell malignancies. Biomed Res Int. 2016;2016:9513037. 27294145 10.1155/2016/9513037 PMC4884835

[45] Jabłońska E , Białopiotrowicz E , Szydłowski M , et al. DEPTOR is a microRNA‐155 target regulating migration and cytokine production in diffuse large B‐cell lymphoma cells. Exp Hematol. 2020;88:56‐67. 32702393 10.1016/j.exphem.2020.07.005

[46] Pachathundikandi SK , Tegtmeyer N , Arnold IC , et al. T4SS‐dependent TLR5 activation by helicobacter pylori infection. Nat Commun. 2019;10:5717. 31844047 10.1038/s41467-019-13506-6 PMC6915727

[47] Tegtmeyer N , Neddermann M , Lind J , et al. Toll‐like receptor 5 activation by the CagY repeat domains of helicobacter pylori. Cell Rep. 2020;32:108159. 32937132 10.1016/j.celrep.2020.108159

[48] Elton TS , Selemon H , Elton SM , Parinandi NL . Regulation of the MIR155 host gene in physiological and pathological processes. Gene. 2013;532:1‐12. 23246696 10.1016/j.gene.2012.12.009

[49] Ohmae T , Hirata Y , Maeda S , et al. Helicobacter pylori activates NF‐kappaB via the alternative pathway in B lymphocytes. J Mmunol. 2005;175(11):7162‐7169. 10.4049/jimmunol.175.11.7162 16301619

[50] Dybkær K , Bøgsted M , Falgreen S , et al. Diffuse large B‐cell lymphoma classification system that associates normal B‐cell subsets phenotypes with prognosis. J Clinical Oncology. 2015;33(12):1379‐1388. 10.1200/JCO.2014.57.7080 PMC4397280 25800755

[51] Hwang J , Suh CH , Won Kim K , et al. The incidence of epstein‐barr virus‐positive diffuse large B‐cell lymphoma: a systematic review and meta‐analysis. Cancers (Basel). 2021;13:1785. 33917961 10.3390/cancers13081785 PMC8068359

