# Supplementary material for: miR‐155‐regulated mTOR and Toll‐like receptor 5 in gastric diffuse large B‐cell lymphoma
Source: Cancer Med. 2021 Dec 16;11(3):555–70. doi: 10.1002/cam4.4466 (PMC8817081; doi:10.1002/cam4.4466)
Supplement: Supplementary file 1 — Fig S1 [file CAM4-11-555-s001.pdf]

Figure S1: Ten of 16 gastric diffuse large B-cell lymphomas could not be classified into the GCB or ABC subtypes

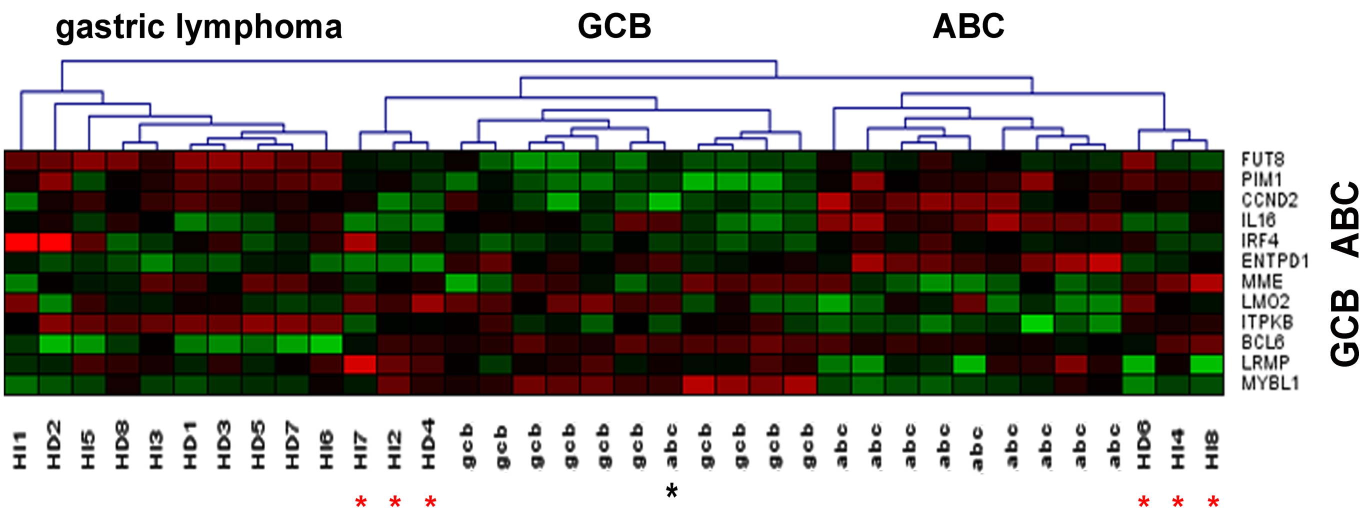

Clustering of our gastric DLBCLs into GCB and ABC types, using as controls, NCBI GEO GSE56315. The classification was based on 6 ABC markers (FUT8, PIM1, CCND2, IL16, IRF4 or Mum1, ENTPD1) and 6 GCB markers (MME or CD10, LMO2, ITPKB, BCL6, LRMP, MYBL1), including the 3 markers, BCL6, Mum1, and CD10, of the Hans system.

For the controls, 10 GCB DLBCLs and 9 of 10 ABC DLBCLs were correctly classified, except one indicated by the black asterisk.

Out of 8 sensitive (HD) and 8 resistant (HI) gastric DLBCLs, most cases could not be appropriately classified, only 3 cases as the GCB type and 3 cases as the ABC type, as indicated by red asterisks.

In fact, the 10 unclassified gastric DLBCLs had a distinctive signature. For example, they had strong expression of the ABC markers FUT8 and PIM1, and the GCB marker ITPKB. The co-expression of ABC and GCB markers, or mix-up of the AGC and GCB markers, is an intrinsic signature, not a statistical artifact, and is distinct for gastric DLBCL, not found in nodal DLBCL.
