# Supplementary material for: miR‐155‐regulated mTOR and Toll‐like receptor 5 in gastric diffuse large B‐cell lymphoma
Source: Cancer Med. 2021 Dec 16;11(3):555–70. doi: 10.1002/cam4.4466 (PMC8817081; doi:10.1002/cam4.4466)
Supplement: Supplementary file 2 — Table S1 [file CAM4-11-555-s004.docx]

| **Table S1 mRNAs suppressed by miR-155 transfected into in BJAB cells** | | | | |
| --- | --- | --- | --- | --- |
| **LK** | **155** | **155/LK** | **Gene** | **Description** |
| 244 | 4 | 0.02 | ABI3BP | cDNA DKFZp667H216 [AL833204] |
| 286 | 3 | 0.01 | ACVR1C | activin A receptor, type IC, transcript variant 1, [NM_145259] |
| 197 | 37 | 0.19 | AQP6 | aquaporin 6, kidney specific, [NM_001652] |
| 147 | 27 | 0.18 | COPZ2 | coatomer protein complex, subunit zeta 2, [NM_016429] |
| 163 | 49 | 0.30 | CYP21A2 | cytochrome P450, family 21, subfamily A, polypeptide 2, transcript variant 2, [NM_001128590] |
| 396 | 84 | 0.21 | DEPTOR | DEP domain containing MTOR-interacting protein, transcript variant 1, [NM_022783] |
| 191 | 59 | 0.31 | DPP9 | dipeptidyl-peptidase 9, [NM_139159] |
| 328 | 33 | 0.10 | EGLN3 | egl-9 family hypoxia-inducible factor 3, [NM_022073] |
| 732 | 146 | 0.20 | F8 | coagulation factor VIII, procoagulant component, transcript variant 1, [NM_000132] |
| 839 | 143 | 0.17 | FCGR2A | Fc fragment of IgG, low affinity IIa, receptor (CD32), transcript variant 2, [NM_021642] |
| 169 | 32 | 0.19 | GRK1 | G protein-coupled receptor kinase 1, [NM_002929] |
| 288 | 52 | 0.18 | ICA1L | islet cell autoantigen 1,69kDa-like, transcript variant 5, [NM_001288624] |
| 284 | 3 | 0.01 | KCNC1 | potassium channel, voltage gated Shaw related subfamily C, member 1, transcript variant 2, [NM_004976] |
| 195 | 24 | 0.12 | KIF26A | kinesin family member 26A, [NM_015656] |
| 192 | 7 | 0.04 | KRTAP5-3 | keratin associated protein 5-3, [NM_001012708] |
| 439 | 4 | 0.01 | NUDT10 | nudix (nucleoside diphosphate linked moiety X)-type motif 10, [NM_153183] |
| 610 | 5 | 0.01 | PIGK | phosphatidylinositol glycan anchor biosynthesis, class K |
| 418 | 12 | 0.03 | RBPMS | cDNA FLJ32971 fis, clone TESTI2008847, [AK057533] |
| 262 | 4 | 0.01 | SCNN1A | sodium channel, non voltage gated 1 alpha subunit, transcript variant 1, [NM_001038] |
| 793 | 6 | 0.01 | TAS2R20 | taste receptor, type 2, member 20, [NM_176889] |
| 334 | 29 | 0.09 | UGT2B15 | UDP glucuronosyltransferase 2 family, polypeptide B15, [NM_001076] |
| 247 | 12 | 0.05 | WDR7 | WD repeat domain 7 |

Filter criteria: LK > 2 times median, 155/LK <0.33, from Supplemental data S2. Note that the filter criteria were set for transcripts with levels higher than the median, because measurements for transcripts with low expression levels were less reliable. Changes in the filter criteria would affect the number of potential targets, but would not affect the final conclusion.
