# Supplementary material for: miR‐155‐regulated mTOR and Toll‐like receptor 5 in gastric diffuse large B‐cell lymphoma
Source: Cancer Med. 2021 Dec 16;11(3):555–70. doi: 10.1002/cam4.4466 (PMC8817081; doi:10.1002/cam4.4466)
Supplement: Supplementary file 3 — Table S2 [file CAM4-11-555-s002.docx]

|  | | | **Table S2 mRNAs suppressed by miR-200 transfected into in U2932 cells** | | | | | | | | |  |
| --- | --- | --- | --- | --- | --- | --- | --- | --- | --- | --- | --- | --- |
| **200a** | **200b** | **200c** | | **LK** | **200a/LK** | **200b/LK** | **200c/LK** |  | **Gene** | | **Description** | |
| 41 | 65 | 72 | | 1287 | 0.03 | 0.05 | 0.06 |  | AICDA | activation-induced cytidine deaminase, [NM_020661] | |  |
| 2041 | 1187 | 2668 | | 8757 | 0.23 | 0.14 | 0.31 |  | AIM2 | absent in melanoma 2, [NM_004833] | |  |
| 1954 | 1722 | 1739 | | 8741 | 0.22 | 0.20 | 0.20 |  | AIRE | autoimmune regulator, transcript variant 2, [NM_000658] | |  |
| 149 | 172 | 166 | | 575 | 0.26 | 0.30 | 0.29 |  | B3GNT7 | UDP-GlcNAc:betaGal beta-1,3-N-acetylglucosaminyltransferase, [NM_145236] | |  |
| 89 | 93 | 113 | | 348 | 0.26 | 0.27 | 0.33 |  | C1QB | complement component 1, q subcomponent, B chain, [NM_000491] | |  |
| 85 | 84 | 83 | | 322 | 0.26 | 0.26 | 0.26 |  | CACNA1B | calcium channel, voltage-dependent, N type, alpha 1B subunit | |  |
| 44 | 55 | 40 | | 453 | 0.10 | 0.12 | 0.09 |  | CCDC24 | coiled-coil domain containing 24, [NM_152499] | |  |
| 37 | 51 | 101 | | 463 | 0.08 | 0.11 | 0.22 |  | CCL4 | chemokine (C-C motif) ligand 4 (CCL4), transcript variant 1, [NM_002984] | |  |
| 49 | 59 | 104 | | 742 | 0.07 | 0.08 | 0.14 |  | CEBPA | CCAAT/enhancer binding protein (C/EBP), alpha, [NM_004364] | |  |
| 318 | 251 | 599 | | 1827 | 0.17 | 0.14 | 0.33 |  | COL27A1 | collagen, type XXVII, alpha 1 | |  |
| 78 | 121 | 135 | | 1059 | 0.07 | 0.12 | 0.13 |  | CXCL10 | chemokine (C-X-C motif) ligand 10, [NM_001565] | |  |
| 79 | 74 | 82 | | 1206 | 0.07 | 0.06 | 0.07 |  | CXCL9 | chemokine (C-X-C motif) ligand 9, [NM_002416] | |  |
| 148 | 211 | 202 | | 1027 | 0.14 | 0.21 | 0.20 |  | EME2 | essential meiotic endonuclease 1 homolog 2 (S. pombe), [NM_001010865] | |  |
| 96 | 99 | 100 | | 730 | 0.13 | 0.14 | 0.14 |  | GTF2IRD2B | GTF2I repeat domain containing 2B | |  |
| 561 | 311 | 2785 | | 8638 | 0.07 | 0.04 | 0.32 |  | IFI44L | interferon-induced protein 44-like, [NM_006820] | |  |
| 1992 | 1188 | 8258 | | 35518 | 0.06 | 0.03 | 0.23 |  | IFITM1 | Homo sapiens interferon induced transmembrane protein 1, [NM_003641] | |  |
| 2626 | 2021 | 6032 | | 21529 | 0.12 | 0.09 | 0.28 |  | IFITM3 | interferon induced transmembrane protein 3, [NM_021034] | |  |
| 130 | 114 | 150 | | 811 | 0.16 | 0.14 | 0.19 |  | LGALS9 | lectin, galactoside-binding, soluble, 9 (LGALS9), transcript variant 1, [NM_009587] | |  |
| 484 | 411 | 2087 | | 11338 | 0.04 | 0.04 | 0.18 |  | LGALS9C | lectin, galactoside-binding, soluble, 9C, [NM_001040078] | |  |
| 30 | 34 | 130 | | 411 | 0.07 | 0.08 | 0.32 |  | LY6E | lymphocyte antigen 6 complex, locus E (LY6E), transcript variant 1, [NM_002346] | |  |
| 48 | 44 | 58 | | 533 | 0.09 | 0.08 | 0.11 |  | NRXN2 | neurexin 2, transcript variant alpha-2, [NM_138732] | |  |
| 67 | 12 | 102 | | 1092 | 0.06 | 0.01 | 0.09 |  | PTGS1 | prostaglandin-endoperoxide synthase 1, transcript variant 1, [NM_000962] | |  |
| 11 | 8 | 24 | | 264 | 0.04 | 0.03 | 0.09 |  | REXO1L1 | REX1, RNA exonuclease 1 homolog (S. cerevisiae)-like 1, [NM_172239] | |  |
| 15 | 42 | 19 | | 266 | 0.06 | 0.16 | 0.07 |  | SAMD12 | sterile alpha motif domain containing 12, transcript variant 1, [NM_001101676] | |  |
| 39 | 9 | 108 | | 435 | 0.09 | 0.02 | 0.25 |  | SELL | selectin L, transcript variant 1, [NM_000655] | |  |
| 62 | 8 | 115 | | 541 | 0.11 | 0.02 | 0.21 |  | SLCO4A1 | solute carrier organic anion transporter family, member 4A1, [NM_016354] | |  |
| 62 | 94 | 64 | | 375 | 0.17 | 0.25 | 0.17 |  | ST3GAL4 | weakly similar to H.sapiens mRNA for Gal-beta(1-3/1-4)GlcNAc alpha-2.3-sialyltransferase. [AK021929] | |  |
| 67 | 115 | 158 | | 808 | 0.08 | 0.14 | 0.20 |  | STRA8 | stimulated by retinoic acid gene 8 homolog (mouse), [NM_182489] | |  |
| 163 | 101 | 166 | | 2560 | 0.06 | 0.04 | 0.07 |  | TMPRSS4 | transmembrane protease, serine 4, transcript variant 1, [NM_019894] | |  |
| 18 | 18 | 20 | | 283 | 0.07 | 0.06 | 0.07 |  | ZNF431 | zinc finger protein 431, [NM_133473] | |  |

Filter criteria: LK>median and 200/LK <0.33 for 200 a, b, & c, from Supplemental data S3. Note that the filter criteria were set for transcripts with levels higher than the median, because measurements for transcripts with low expression levels were less reliable. Changes in the filter criteria would affect the number of potential targets, but would not affect the final conclusion.
