# Supplementary material for: miR‐155‐regulated mTOR and Toll‐like receptor 5 in gastric diffuse large B‐cell lymphoma
Source: Cancer Med. 2021 Dec 16;11(3):555–70. doi: 10.1002/cam4.4466 (PMC8817081; doi:10.1002/cam4.4466)
Supplement: Supplementary file 4 — Data S1‐S4 [file CAM4-11-555-s003.docx]

**Supplementary data SD1: Genome-wide miRNA profiles in gastric diffuse large B-cell lymphomas**

A series of nine *H. pylori* eradication therapy-sensitive and eight *H. pylori* eradication therapy-resistant gastric DLBCLs was used. RNAs were extracted from sections of the biopsy specimen taken at the time of initial diagnosis. The nCounter miRNA Expression Analysis System (NanoString Technologies, Seattle, WA) was used to obtain the expression profiles of 654 human miRNAs. The data are submitted as GEO Super-series GSE182362.

**Supplementary data SD2: Genome-wide mRNA profiles in miR-155-BJAB**

BJAB cells transfected with a miR-155-expressing vector were established. Genome-wide mRNA profiles were performed with Agilent-072373 SurePrint G3 Human GE 8x60K Microarray (NCBI GEO platform GPL21185), according to the manufacturer’s recommendations (Agilent Technologies, Santa Clara, USA). The data are submitted as GEO Super-series GSE182362.

**Supplementary data SD3: Genome-wide mRNA profiles in miR-200 a, b, or c-U2932**

Three U2932 cell lines, transfected with an expressing vector for miR-200 a, b, or c, respectively, were established. Genome-wide mRNA profiles were performed with Agilent-028004 SurePrint G3 Human GE 8x60K Microarray (NCBI GEO platform GPL14550), according to the manufacturer’s recommendations (Agilent Technologies, Santa Clara, USA). The normalized data are submitted. The data are submitted as GEO Super-series GSE182362.

**Supplementary data SD4: Genome-wide mRNA profiles in gastric diffuse large B-cell lymphomas**

A series of eight *H. pylori* eradication therapy-sensitive and eight *H. pylori* eradication therapy-resistant gastric DLBCLs was used. RNAs were extracted from the biopsy specimen taken at the time of initial diagnosis. Agilent-039494 SurePrint G3 Human GEv2 8x60K Microarray (NCBI GEO platform GPL17077) was used according to the manufacturer’s recommendations (Agilent Technologies, Santa Clara, USA). The data are submitted as GEO Super-series GSE182362.
